# Impact of cell wall encapsulation of almonds on *in vitro* duodenal lipolysis

**DOI:** 10.1016/j.foodchem.2015.04.013

**Published:** 2015-10-15

**Authors:** Myriam M.L. Grundy, Peter J. Wilde, Peter J. Butterworth, Robert Gray, Peter R. Ellis

**Affiliations:** a King’s College London, Diabetes and Nutritional Sciences Division, Biopolymers Group, Franklin-Wilkins Building, London SE1 9NH, UK; b Institute of Food Research, Norwich Research Park, Colney, Norwich NR4 7UA, UK

**Keywords:** DAG, diacylglycerol, FFA, free fatty acids, GI, gastrointestinal, L, linoleic acid, MAG, monoacylglycerol, NaGDC, sodium glycodeoxycholate, NaTC, sodium taurocholate hydrate, O, oleic acid, P, palmitic acid, SEM, standard error of the mean, TAG, triacylglycerol, Lipid bioaccessibility, Almond, Lipolysis, Plant cell walls, Encapsulation

## Abstract

Although almonds have a high lipid content, their consumption is associated with reduced risk of cardiovascular disease. One explanation for this paradox could be limited bioaccessibility of almond lipids due to the cell wall matrix acting as a physical barrier to digestion in the upper gastrointestinal tract. We aimed to measure the rate and extent of lipolysis in an *in vitro* duodenum digestion model, using raw and roasted almond materials with potentially different degrees of bioaccessibility. The results revealed that a decrease in particle size led to an increased rate and extent of lipolysis. Particle size had a crucial impact on lipid bioaccessibility, since it is an indicator of the proportion of ruptured cells in the almond tissue. Separated almond cells with intact cell walls showed the lowest levels of digestibility. This study underlines the importance of the cell wall for modulating lipid uptake and hence the positive health benefits underlying almond consumption.

## Highlights

• Tissue microstructure controlled rate and extent of *in vitro* lipolysis in almonds.
• Lipolysis methods using pH-stat and GC analysis were in good agreement.
• Increasing lipid bioaccessibility led to increased levels of digestibility.
• Almond cell walls restrict lipid release, thus hindering digestion kinetics.

## 1 Introduction

Plant foods, such as lipid-rich almond seeds are complex matrices with structures ranging in scale, from the cm dimensions of plant tissue to the nm scale of nutrient molecules inside plant cells. In order to be digested in the human stomach and small intestine, intracellular lipids have to be released from the tissue and emulsified to increase susceptibility of the lipid to lipase action. Previously, it was assumed, by many researchers, that most of the lipid and other macronutrients are released during mastication and available for digestion and absorption in the upper gastrointestinal (GI) tract (Bauer, Jakob, & Mosenthin, 2005). However, it has been known for some time that the digestion of nutrients from various edible plants can vary substantially and that food structure and properties are important factors in explaining this variation (Parada & Aguilera, 2007). For instance, the cell walls (i.e. the main source of dietary fibre) of almonds and other plant foods can behave as physical barriers that hinder the bioaccessibility and hence digestion of entrapped lipid (Ellis et al., 2004). Indeed the role of cell walls, in regulating the bioaccessibility (release) of lipid and other nutrients in edible plants, has received considerable attention (Ellis et al., 2004; Tydeman et al., 2010; Grundy et al., 2015). Bioaccessibility is defined, in this paper, as the proportion of a nutrient “released” from a complex food matrix and, therefore, potentially available for digestion and/or absorption in the GI tract.

The composition of almond seeds varies according to a number of factors, including the variety and the harvest; but, typically, the seeds contain approximately 50% of lipids and 12% of dietary fibre, which is mainly derived from cell wall polysaccharides (Yada, Lapsley, & Huang, 2011). The lipid components of the seeds are mainly located in intracellular oil-bodies in the form of triacylglycerol (TAG) (Ellis et al., 2004), the predominant fatty acids of which are oleic, linoleic and palmitic. The oil-bodies have an average diameter of 2–3 μm, approximately, and are surrounded by a single layer of phospholipids in which proteins, mainly oleosins, are embedded (Beisson, Ferte, Voultoury, & Arondel, 2001).

Recent studies have provided evidence to show why a cell wall barrier mechanism impairs the bioaccessibility and extent of digestion of lipid in almonds, despite their status as a high energy food (Novotny, Gebauer, & Baer, 2012; Grassby et al., 2014; Mandalari et al., 2014; Grundy et al., 2015). The presence of intact cell walls from almond tissue during digestion also has a significant influence on postprandial lipaemia, as shown by Berry et al. (2008). They reported that muffins made with large almond particles, comprising mainly intact cells with encapsulated lipid, elicited a lower lipaemic response in healthy human subjects than did the muffins containing defatted almond flour and free almond oil. Furthermore, the results of our recent mastication study showed that 35–40% of the almond bolus was composed of particles with a size superior to ∼500 μm (Grundy et al., 2015), suggesting that the particles reaching the stomach probably contain a significant proportion of intact cells with encapsulated lipid. Also, negligible changes in structure and particle size seem to occur during *in vitro* gastric digestion (Mandalari et al., 2014). Thus we concluded that lipid present on the surface of the almond particles is available for hydrolysis by lipase, whereas the intracellular lipid located in intact cells is largely unavailable and remains undigested in the early stages of digestion (Mandalari et al., 2008, 2014; Grundy et al., 2015).

The purpose of the present study was to determine the effect of varying the proportion of ruptured/intact cells on the rate and extent of *in vitro* lipid digestion (expressed as the amount of free fatty acids released) in raw and roasted almond materials. The samples were manipulated such that they exhibited marked differences in lipid bioaccessibility, and included ground and chewed particles of almond and also intact almond cells. To address this main objective, two methods were employed to measure the free fatty acids release: the pH-stat titrimetric method and gas chromatography (GC) analysis. As far as we are aware, however, this is the first time that the pH-stat method has been used to study the effects of lipid bioaccessibility on lipolysis. This study focussed on simulating the digestion in the duodenal compartment only, because the majority of lipid hydrolysis is considered to take place in the duodenum (Bauer et al., 2005).

## 2 Materials and methods

### 2.1 Materials

Natural raw and roasted almonds (*Amygdalus communis* L.; variety Nonpareil) were produced by Hughson Nut and supplied by the Almond Board of California. The roasted almonds were produced by using a standardised method of hot air (dry) roasting (150 °C for 15 min). Powdered beta-lactoglobulin (β-Lg) was donated by Davisco Foods International (JE 002-8-415, Le Sueur, USA). Almond oil, glyceryl tributyrate (99%), glyceryl trioleate (65%), sodium dihydrogen phosphate (99%), disodium hydrogen phosphate (99%), trans-1,2-diaminocyclohexane-N,N,N′,N′-tetraacetic acid (CDTA, 98.5%), sodium metabisulphite (⩾99%), sodium chloride (99.5%), calcium chloride (93%), sodium glycodeoxycholate (NaGDC, ⩾97% TLC) and pancreatin from porcine pancreas (L3126, activity 100–400 units/mg protein, where 1 unit corresponds to 1 micro-equivalent of fatty acid released from olive oil in 1 h at pH 7.7, 37 °C) were purchased from Sigma–Aldrich Chemical Co. (Poole, UK). The oil of roasted almond was obtained from Huilerie Croix Verte (Neuillé, France) and sodium taurocholate hydrate (NaTC, ⩾97% TLC) was obtained from Alpha Aesar (Ward Hill, USA). Internal standards for gas chromatography analysis, C15:0 (pentadecanoic acid, monopentadecanoin, 1,3-dipentadecanoin, tripentadecanoin), were purchased from Nu-Chek-Prep, Inc (Elysian, USA).

### 2.2 Emulsion preparation and characterisation

Almond oil emulsion was included as a reference sample with a high lipid bioaccessibility (100%). β-Lg solution was prepared by dissolving 1 wt% of powdered β-Lg in 10 mM phosphate buffer (pH 7.0 at 37 °C) and stirring for at least 2 h. Emulsions were made from either synthetic lipids commonly used to determine lipase activity, namely, tributyrin and triolein, or almond oil. Raw and roasted almond oils contained approximately 64.1% and 63.1% of oleic acid (O), 26.1% and 25.9% of linoleic acid (L), and 6.8% and 7.2% of palmitic acid (P), respectively (analysis performed by gas liquid chromatography as described below). The emulsions were obtained by pre-emulsifying 1.6 wt% of oil in β-Lg solution using a homogeniser (Ultra-Turrax T25, IKA® Werke, from Fisher Scientific Ltd.) for 1 min at 1100 rpm. The pre-emulsion was then sonicated with an ultrasonic processor (Sonics & Materials Inc, Newtown, USA) at 70% amplitude for 2 min. The lipolysis of unemulsified oils, for raw and roasted almond samples, as shown in Section 3, was performed by dispersing the oils directly into the reaction vessel without preliminary preparation.

The droplet size distributions of the emulsions were measured with a laser light scattering instrument (Beckman Coulter LS13320®, Beckman Coulter Ltd., High Wycombe, UK). Water was used as a dispersant (refractive index of 1.330), and the absorbance value of the oil droplets was 0.001. Almond oil has a refractive index of 1.471, tributyrin 1.435 and triolein 1.470, as measured using a refractometer (Rhino Brix90 Handheld Refractometer, Reichert, Inc., New York, USA). The particle size measurements were reported as the average volume diameter: $d_{4\text{,}3}=\sum n_{i}d_{i}^{4}/\sum n_{i}d_{i}^{3}$, where *n_i_* is the number of droplets of diameter *d_i_*. Values are presented as the means ± SEM of at least three replicates.

### 2.3 Preparation of almond samples

Almond cells were separated by soaking 2–3 mm almond particles for 4 weeks, with rotation, in a solution containing a chelating agent (50 mM CDTA) and a preservative (5 mM Na_2_S_2_O_5_) at pH 7.0 (Mandalari et al., 2014). The samples were briefly rinsed and then mashed using a mortar and pestle to a paste consistency. The almond pieces were loaded onto a sieve of 53 μm and a 20 μm nylon mesh, as well as a sieve base to collect the liquid. After elimination of most of the water, the material present on the nylon mesh was then transferred into a dialysis membrane (Float-A-Lyzer G2 10 ml, 3.5–5 Kd, Sigma). The membrane was placed in phosphate buffer (10 mM, pH 7.0) for about 4 h; the operation was repeated 4 times as recommended by the manufacturer. The dialysis permitted the removal of CDTA from the separated cells, which is important since CDTA is known to inhibit lipase activity (Weaber, Freedman, & Eudy, 1971).

Almond particles of four different size ranges (1000–2000, 500–1000, 250–500, and <250 μm) were obtained by grinding raw and roasted almonds in a blender (E825bk, Lloytron PLC, Leigh, UK) before sieving and collecting the particles from sieves of 1000, 500 and 250 μm aperture, as well as a sieve base (size < 250 μm).

Masticated samples (triplicate boluses) were obtained, following a protocol described in detail elsewhere (Grundy et al., 2015). This study was approved by the Research Ethic Committee of the North London’s National Research Ethics Service (Reference No. 10/H0717/096) and registered at isrctn.org as ISRCTN58438021. Predicted values of lipid (mainly TAG) bioaccessibility of the almond samples were obtained from our theoretical model, using average particle size data (Grassby et al., 2014; Grundy et al., 2015).

### 2.4 *In vitro* duodenal digestion (pH-stat)

The pH-stat method is a rapid and convenient tool to study lipolysis occurring in the duodenal compartment and on synthetic lipids (e.g. tributyrin and triolein) and olive oil (Beisson, Tiss, Riviere, & Verger, 2000). This method has been widely used and previous investigations have not been restricted to pancreatic lipase only, since the activity of gastric lipase has also been studied (McClements & Li, 2010). The rates of lipolysis were continuously measured by titration of released free fatty acids (FFA) with 0.15 M NaOH at 37 °C and an endpoint of pH 7.0. Each assay was performed over 1 h in a mechanically stirred reaction vessel of a pH-stat instrument (Titrino 848 plus, Methrohm UK Ltd.). The *in vitro* duodenal digestion model used was adapted from previous studies (Li & McClements, 2010; Li, Hu, & McClements, 2011) with a reaction medium as follows: (i) 19 ml of sample containing 300 mg of lipids, in the form of either emulsion, separated almond cells or almond particles, dissolved in β-Lg solution; (ii) 15 ml of bile salt solution (31.5 mM in 10 mM phosphate buffer, pH 7.0, 37 °C); (iii) 1 ml of NaCl (5.63 M in deionised water) and 1 ml of CaCl_2_ (0.375 M in deionised water). The system was adjusted to pH 7.0 with 0.15 M NaOH and then 1.5 ml of freshly prepared lipase solution were added (40 mg/ml in 10 mM phosphate buffer). The volume of the reaction system in the vessel was 37.5 ml and its final composition was 0.8 wt% lipid, 12.5 mM bile salts, 2.4 mg/ml of lipase, 150 mM NaCl and 10 mM CaCl_2_.

As a control, the pH fluctuation of the assay mixture alone was determined by running the titration without any lipase; the volume obtained was then deducted from the volume data produced from the subsequent test lipid samples. This step of the titration was repeated for each sample and it ensured the accurate quantification of newly formed FFA derived from the test samples. Since the modelled duodenal digestion conditions were the same for all materials, primarily the amount of lipid, a difference in lipolysis rates would indicate that there were differences in lipid bioaccessibilities among those materials. Each digestion reaction was repeated at least three times.

The volume of NaOH solution (in ml), added as a function of time to keep the pH constant, is equivalent to the amount of FFA released. Also, since the hydrolysis of TAG leads to one molecule of monoacylglycerol (MAG) and two of FFA, the percentage of FFA released can be calculated as follows:

(1) $$\%\;\text{FAA}=100\times \left(\frac{V_{\text{NaOH}}\times m_{\text{NaOH}}\times M_{\text{lipid}}}{w_{\text{lipid}}\times 2}\right)$$

where *V*_NaOH_ corresponds to the volume of NaOH required to neutralise the FFA produced, *m*_NaOH_ is the concentration of the NaOH solution used (in M), *w*_lipid_ is the total mass of TAG initially present in the reaction vessel (in g), and *M*_lipid_ is the molecular weight of oil (in g/mol) (Li & McClements, 2010). The molecular weight of almond oil was estimated to be 878 g/mol; this value was calculated from the TAG composition of the oil and the occurrence of the FFA (O, L, P) within these TAGs (Holcapek, Jandera, Zderadicka, & Hruba, 2003). The initial rates of lipolysis were calculated from the slopes of the amount of product (in μmol) versus time (in minutes) plots.

### 2.5 Lipid analysis by GC

Digestion of almond materials was performed in the pH-stat, as described in Section 2.4; a separate experiment was performed for each reaction with sampling times of: 0, 2, 5, 15, 20 and 30 min following initiation of the reaction. The lipids present in the aqueous phase of the samples (i.e. extracellular lipids in digested samples of almond particles and cells) were extracted from the reaction vessel at the different time points, using a 2:1 chloroform – methanol (v/v) solution containing C15 internal standards (Folch, Lees, & Sloane Stanley, 1957). The mixture was centrifuged at 425 g for 5 min at 4°C and then 400 μl of the chloroform layer was collected and transferred into test tubes. The samples were evaporated to dryness in a heated centrifugal evaporator (Genevac SF50, Genevac Ltd., Ipswich, UK) connected to a pump (Büchi Vac®V-500, Büchi Labortechnik AG, Flawil, Switzerland) for approximately 45–60 min. The dried extracts were dissolved in 50 μl of chloroform and the different classes of lipids were separated by solid phase extraction, based on a method previously described (Ruiz, Antequera, Andres, Petron, & Muriel, 2004). The separation was performed using a 20-port vacuum manifold coupled to a vacuum pump and LRC cartridges (Agilent HF Bond Elut LRC-NH2, Agilent Technologies UK Ltd., Wokingham, UK). All neutral lipids (TAG, DAG & MAG) were eluted with a solution of chloroform – isopropanol (2:1, v/v) and the FFA with a chloroform – methanol - acetic acid (100:2:2, v/v) solution.

Each fraction was evaporated to dryness in the heated centrifugal evaporator. Fatty acid methyl esters (FAME) were obtained by dissolving each sample in 1.8 ml of a solution containing methanolic HCl (3 M) and toluene (80:20, v/v), based on a method published by Lepage and Roy (1986). The tubes were placed in the incubator at 40 °C overnight. Following cooling, the reaction mixture was neutralised by addition of 5 ml of 6% (w/v) sodium carbonate. The toluene layer (containing the FAME) was collected and dispersed into chromatography vials. FAME were quantified, using gas chromatography (7890A, Agilent Technologies UK Ltd, Wokingham, UK) equipped with an on-column injector port (BPX70, SGE Europe Ltd, Milton Keynes, UK) and flame ionisation detector. The temperature programme started at 100 °C for 4 min, increased to 200 °C in 1 min, held for 6 min, then increased to 240 °C in 1 min with a final hold time of 5 min. The fatty acids were identified and quantified by comparing their relative retention times with those of standards. The concentration of FFA release was determined, using the C15 internal standards, and expressed in micromolar (μM) terms.

### 2.6 Microstructural analysis

The structural changes of the separated lipid-rich cells, pre- and post-digestion, were studied, using either optical (Zeiss Axioskop 2 mot plus, Carl Zeiss Ltd, UK) or confocal laser scanning (CLSM; Leica TCS SP2, DMIRE2 inverted, Milton Keynes, UK) microscopes, with 40× (N.A. 1.25) and 63× (N.A. 1.32) oil immersion objective lenses. For optical microscopy, samples of almond cells (unstained) were either suspended in buffer (undigested samples) or collected from the reaction vessel (digested samples), and then mounted on a glass slide, covered and viewed immediately. Cells observed with CLSM were stained with Nile red (1 mg/ml in dimethyl sulphoxide). The samples were excited with an argon laser at 488 nm, and the fluorescence emitted by the samples was detected at 630–680 nm.

### 2.7 Statistical analysis

The data were analysed, using SPSS version 17.0. For all tests, the significance level was set at *P* < 0.05 (2 tailed). Percentages of FFA release, fatty acid concentrations and reaction rates were assessed by repeated-measures analysis of variance (ANOVA) with time and materials (i.e. emulsion, cells and particles) as ‘within-sample’ factors. Differences between raw and roasted samples were analysed by Student’s paired *t*-test. The relationship between predicted lipid release, using the theoretical model and percentages of FFA release, was analysed by regression analysis.

## 3 Results and discussion

### 3.1 Lipolysis of almond oil and other oil emulsions by pH-stat

Pancreatic lipase catalyses the hydrolysis of TAG and generates free fatty acids (FFA). The reaction can be monitored by maintaining a constant pH by automated addition of NaOH, which neutralises the newly formed FFA. The volume of NaOH solution thus added corresponds to the amount of FFA released. Values for the extent of digestion were calculated as the amount of FFA produced relative to the total amount of TAG in the sample over 60 min. Digestion results, expressed as a percentage FFA at 60 min, and for the initial reaction rates (μmol/min) are presented in Table 1.

In our experiments, between 67% and 70% of the almond oil emulsion initially present in the reaction vessel was digested in one hour; which accords with the extent of lipid digestion of oils occurring in the human GI tract (Bauer et al., 2005).

**Fig. 1:**
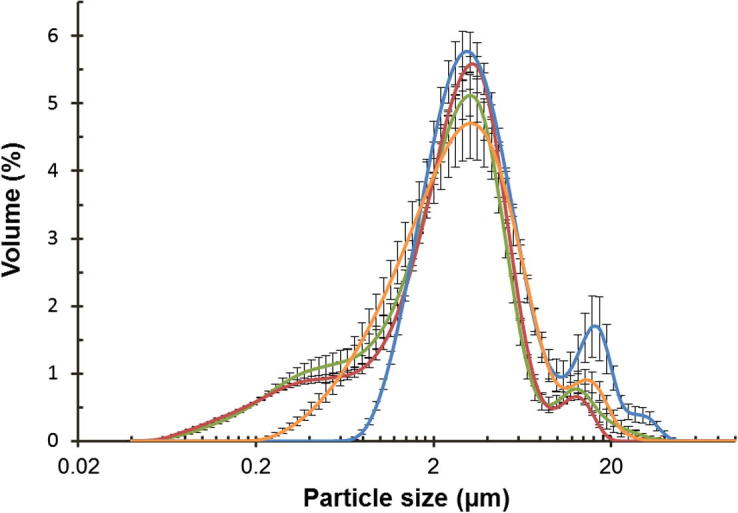
Particle size as percentage volume of raw () and roasted () almond oils, tributyrin (), and triolein () emulsions. Values are presented as means ± SEM (*n* = 3).

The initial rates of reaction, as well as the quantity of FFA produced in 60 min, were greater for emulsions than for non-emulsified oils and this was found regardless of the oil type (Table 1). As previously reported, the rate and extent of lipid digestion were strongly influenced by the structure and composition of the emulsion (Li et al., 2011). Both the digestion rate and its extent increased with a decrease in emulsion droplet size when the oils were emulsified, due to the larger available surface area (smaller mean droplet diameter) in the emulsions, compared with the non-emulsified almond oils, which is in agreement with previous work (Armand et al., 1992). The droplet sizes of the emulsions were found to be similar for raw and roasted almond oils, with mean values (±SEM) of 3.2 ± 0.04 and 3.4 ± 0.27 μm, respectively (Fig. 1). Tributyrin emulsions were composed of droplets of slightly greater mean size (3.9 ± 0.23 μm) than those of the almond oils but, for triolein emulsion, the mean droplet size (2.9 ± 0.07 μm) was slightly lower than that of the almond oils, but significantly lower than that of the tributyrin droplets.

The lipid composition of the emulsion is another key feature that influences lipolysis (Zhu, Ye, Verrier, & Singh, 2013). Indeed, the long chain FFAs (i.e. oleic and linoleic acids) generated from the hydrolysis of triolein and almond oils are relatively water-insoluble and therefore not easily removed from the interface, and need to be micellised by the presence of bile salts. The *in vitro* digestion model is a closed system without an absorption phase to remove excess lipolytic product. It contains a finite concentration of bile salts, capable of “solubilising” a limited quantity of FFA, thus limiting the extent of FFA released. As the ability of the bile salt micelles to “solubilise” FFA diminishes, the interface becomes saturated with FFA, which then inhibits further lipolysis (Golding & Wooster, 2010). The generated MAGs are also strongly interfacial-active molecules; if they are not removed by bile salts, they inhibit lipase activity by “monopolising” the surface of the oil droplet (Reis, Holmberg, Watzke, Leser, & Miller, 2009). In contrast, the lipolysis product of tributyrin (butyric acid) has a much lower molecular weight than has the FFA released from the other oils, and is highly soluble in water; therefore, this product does not require the presence of bile, and will partition directly into the aqueous phase until its solubility limit is reached (Borgström, 1967). Hence the smaller amounts of FFA released from almond and triolein, compared with those generated from tributyrin lipolysis, are probably explained by product inhibition at the water–oil interface. Since triolein and almond oils showed equivalent degrees of lipolysis, it seems that the presence of linoleic acid in almond oils did not influence the course of the reaction and this was observed despite the slight difference in droplet sizes between the two emulsions. This result is consistent with a previous *in vitro* digestion study showing that, for food oils where the fatty acids are composed mainly of C16 and C18 fatty acids, no preferential selection in the type of fatty acids released occurs during lipolysis (Mandalari et al., 2008).

No statistically significant differences were found in either the reaction rate or total FFA production between raw and roasted almond oil emulsions, which was anticipated given that the fatty acid compositions of the emulsions were similar. Also, these results are not unexpected, given that the characterisation of the almond oil showed that the roasting process had no effect on droplet size (i.e. surface area to volume ratio).

As lipase is only active at the interface, it was expected that a lag phase would be observed, during which the enzyme adsorbs onto the emulsion surface. The “interfacial quality”, governed notably by the interfacial molecular organisation, including the presence of lipolytic products, and the interfacial conformations of lipids, are highly relevant to the kinetics of the lipase (Reis et al., 2009). In the present study, bile salts and calcium ions were added to the reaction mixture, together with the substrate, prior to the addition of lipase, and any other surface-active molecules should have been removed from the interface, hence facilitating the immediate adsorption of the enzyme at the interface. Therefore no lag phase was observed. Bile salts are unusual surfactants that play a crucial role in lipid digestion and absorption (Maldonado-Valderrama, Wilde, Macierzanka, & Mackie, 2011). The interfacial protein network (e.g. β-Lg) is displaced by bile salts (Maldonado-Valderrama et al., 2008), and the interface (thus covered by bile salts) is known to promote colipase, and subsequently lipase, adsorption. Bile salts are also required to remove the lipolytic products that accumulate at the interface and prevent lipase inhibition. Given that different bile salts exhibit different behaviours at the interface (promoting either colipase/lipase anchoring to the interface or displacement of lipolytic products), it is preferable to use a mixture of bile salts, such as for instance NaTC and NaGDC (Parker, Rigby, Ridout, Gunning, & Wilde, 2014).

### 3.2 Lipolysis of almond cells and particles, using the pH-stat method

**Fig. 2:**
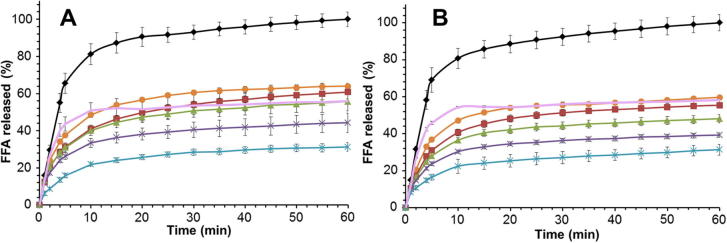
Percentage of free fatty acid (FFA) released versus lipolysis time over 60 min of raw (A) or roasted (B) almond materials prepared with different degrees of lipid bioaccessibility: almond oil emulsion (), almond particles < 250 μm (), 250–500 μm (), 500–1000 μm (), 1000–2000 μm (), separated almonds cells () and chewed almonds (). Values were normalised relative to the almond oil emulsion (100% release at 60 min) and presented as means ± SEM (*n* = 3).

Fig. 2 presents the results of FFA release from almond samples during one hour of incubation. The data were normalised so that the values of the almond particles and cells were calculated as percentage of the almond oil emulsion (i.e. the reference sample). Thus, the maximum amount of FFA produced during the 60 min duodenal digestion of almond oil emulsion was 100%. It would have been anticipated that the lipid droplets, formed by the release of lipid from ground and chewed almond, had interfaces with compositions different from those found in the emulsion droplets, which is highly likely therefore to affect the lipolysis rate. These interfaces of the emulsions will have been composed of β-Lg, whereas the lipids released from the almond particles will be coated with the storage proteins initially present in the almond tissue, plus phospholipids and oleosins that are located on the surface of the oil bodies. However, when the interface is exposed to bile salt solution with a concentration > 5 mM, as is the case in these experiments, the bile salts would have displaced most, if not all, of the adsorbed material. The interface is thus likely to have been dominated by the bile salts and not proteins and/or phospholipids (Maldonado-Valderrama et al., 2008).

For both raw and roasted almonds, lipid digestion was significantly more limited for separated cells, showing ∼31% FFA release over 60 min for both raw and roasted almond cells, relative to the reference emulsion normalised to 100% (see Fig. 2). Lipid digestion was also markedly restricted for the raw and roasted almond particles (from 44–64% to 39–58% FFA release for raw and roasted almond particles, respectively). Furthermore, an inverse relationship between particle size and FFA release was observed (Fig. 2 and Table 2). The predicted lipid bioaccessibility values and FFA release seem to be linearly related (*R*^2^ ≈ 0.65 for both raw and roasted almonds). The higher concentration of FFA generated from smaller particles, compared with the largest particles, is attributed to the greater number of ruptured cells, and therefore increased lipid bioaccessibility, as previously shown (Ellis et al., 2004; Grassby et al., 2014; Mandalari et al., 2014; Grundy et al., 2015). As predicted, the extent and rate of lipolysis were greatest for the particles with the smallest size (⩽250 μm), which correspond to the sample with the largest proportion of ruptured cells on the surfaces of the particles, with lipid content more accessible to the lipase. Despite the significant amount of large particles contained in the chewed almond boluses (35–40% of particles > 500 μm) (Grundy et al., 2015), the initial lipolysis rate was more rapid than that of the milled almond samples of similar average particle size (Table 2). However, this result is less surprising when the broad size distribution of particles of chewed almonds is taken into consideration, as previously reported (Grundy et al., 2015). Thus, although mm sized particles were present in chewed samples, they also contained relatively small-sized particles, ∼50% of which had sizes ⩽ 125 μm, for both raw and roasted almonds. Such small particles contain a greater proportion of bioaccessible (available) lipid relative to larger particles with lower surface/volume ratios and thus give less lipid release. On this basis, we would have expected to observe a similar high initial rate of lipolysis for almond flour (i.e. <250 μm sample, Fig. 2 and Table 2), but both raw and roasted types had significantly lower initial lipolysis rates than had the chewed samples. This suggests that there may be factors, other than particle size, that explain the relatively high rates of lipolysis of masticated almonds. For instance, the method of trituration applied to almonds is likely to affect the physical characteristics of almond particles. The chewing of almond seeds crushes and compresses the almond tissue, resulting in cell damage (rupture) on and beneath the fractured surfaces of mainly small particles, compared with a laboratory blending process employing sharp blades that seem to generate fewer damaged particles with smoother cut surfaces (Grundy et al., 2015). Thus, in masticated almond tissue, there is likely to be more fissuring than that which occurs with mechanical blending, so that the intracellular lipid is more accessible to digestive fluid but, also, the greater degree of compression through chewing may physically force more lipid out of the cells. A higher concentration of lipid at the almond particle surface may explain why chewed particles are closer to the initial hydrolysis rates of almond oil emulsions than are blended samples.

**Fig. 3:**
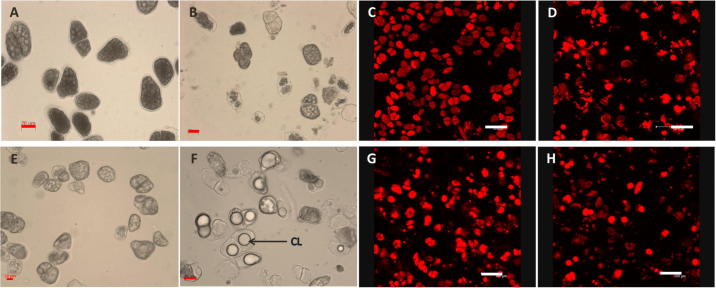
Representative images of separated, raw (A–D) and roasted (E–H) almond cells before (A, C, E and G) and after (B, D, F and H) digestion as examined by optical (A, B, E and F) or confocal (C, D, G and H) microscopy. CL = coalesced lipids. Lipid in C, D, G and H is stained with Nile red. Scale bars: A, B, E and F = 20 μm; C, D, G and H = 100 μm.

No statistically significant differences in the amount of FFA produced over 60 min (Fig. 2, *P* = 0.067) or reaction rate (Table 2, *P* = 0.064) were observed between raw and roasted particles, indicating that thermal processing had negligible effects on digestion kinetics. The micrographs in Fig. 3 clearly show that much of the intracellular lipids still remained encapsulated inside the almond cells after digestion. The changes in cell morphology seemed minimal, except for extensive lipid coalescence in roasted samples, which is consistent with our recent observations (Mandalari et al., 2014). However, some microstructural studies have shown that the roasting of almonds also alters the shape of almond parenchyma cells, ruptures some of the cell walls, produces uneven distribution and coalescence of the oil bodies, and causes some aggregation of the protein bodies (Pascual-Albero, Perez-Munuera, & Lluch, 1998; Varela, Aguilera, & Fiszman, 2008). It is possible, therefore, that roasting may have increased the porosity of the cell wall, thereby allowing greater access of digestive fluid, but did not facilitate significant intracellular lipolysis because of the presence of coalesced lipids (i.e. lower surface area: volume ratio). Larger lipid aggregates can be seen inside the roasted almond cells after digestion (Fig. 3F) suggesting that some transformation had occurred (e.g. by bile salt and/or lipase penetration, and destabilisation of the surface proteins of the oil bodies).

A measurable amount of FFA was produced from the digestion of almond cells (Fig. 2), which is probably due to the cell preparation containing a small proportion of ruptured cells and thus some bioaccessible lipid. Some evidence for this was observed by microscopy (data not shown). Indeed, it is extremely difficult to obtain a preparation that is completely devoid of free lipids and damaged cells. The released lipid in the fractured cells of the preparation would have been susceptible to digestion (hence explaining perhaps the 31% of FFA release observed after 60 min, see Table 2).

For all materials studied here, the reaction rate had virtually plateaued after about 20 min of reaction, indicating that minimal further digestion had occurred. It is possible that the lipase had neither access to the encapsulated lipids nor to the lipids that may have diffused out of the cell. This levelling out of the reaction rate could be attributed to a loss of enzyme activity, and/or product inhibition in the case of digestion of the almond oil emulsions, but this explanation cannot be true for the almond particles or cells, since a much lower amount of FFA was produced from these materials.

### 3.3 A comparison of pH-stat and GC methods of lipolysis analysis

**Fig. 4:**
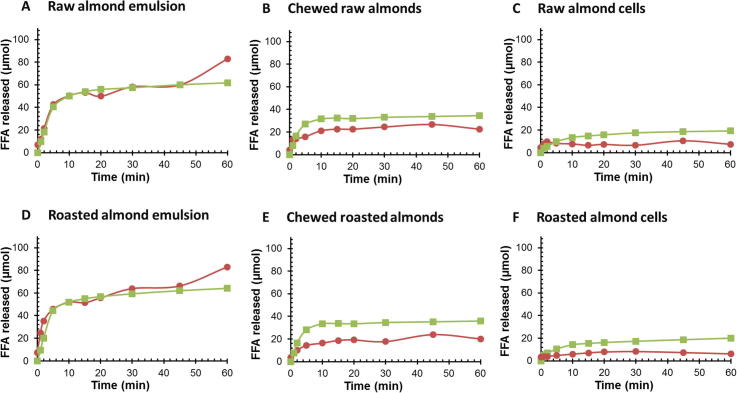
FFA released (μmol) over a 60 min time period during duodenal digestion, using the pH-stat method () and GC analysis (, average values duplicates) for raw (A–C) and roasted (D–F) almonds; almond emulsions (A and D), chewed almonds (B and E) and separated almond cells (C and F).

The amounts of lipolytic products (μmol) generated from the duodenal digestion of raw and roasted almond emulsions, chewed almonds and almond cells, as measured by pH-stat and GC, are presented in Fig. 4. The lipolysis profiles clearly show a difference in FFA release between materials with the lowest values observed for separated cells. In all cases, most of the lipolysis took place in the first 10 min of digestion. Identical trends were observed for both raw and roasted samples. The FFA release data obtained from both the pH-stat and GC methods followed more or less the same trend, although the FFA curves for GC analysis of chewed almonds and almond cells were slightly lower. In a recent study by Helbig, Silletti, Timmerman, Hamer, and Gruppen (2012), using an emulsion only, GC analysis was found to give FFA values 2–3 times higher than values obtained by the pH-stat. The methodology used by the authors was different, however, from that employed by our group, particularly with respect to the GC method. The discrepancy in FFA values between the pH stat and the GC curves observed for the chewed almonds and separated cells possibly reflects a limitation associated with the extraction procedure for the GC preparation. Indeed, it is possible that some of the hydrolysed lipid products were lost during the solid phase extraction and the preparation of the samples for GC analysis. However, GC analysis was used in the present study in order to validate the use of the pH-stat with almond materials (particles and cells). This objective was achieved, given that the two methods followed the same trend, particularly at the beginning of the lipolysis, when most of the lipids are digested. The current study was performed to compare the digestibility of lipids contained in almond materials with various degrees of complexity, using a model simulating the duodenal phase under well-controlled conditions. For this purpose, the pH-stat was the simplest, cheapest and most convenient technique, that permitted continuous measurements of lipolysis.

## 4 Conclusions

The current *in vitro* study showed that, depending on the physical state of the almond materials, modified by mastication and mechanical processing, the availability of lipids for lipolysis, and the amount of fatty acids produced from the hydrolysis of TAG varied significantly. The amount of FFA release was greater for smaller particles than for larger particles, due to the greater proportion of ruptured cells, and therefore the amount of released (free) lipid available for digestion. The marked differences in lipid digestion between almond emulsion and lipid-rich almond particles, including separated cells, confirm the crucial role played by plant cell walls as a physical barrier against nutrient release and digestion. Despite their small size (∼30–35 μm), almond cells gave less FFA release than did almond particles, supporting the idea that lipid bioaccessibility relies not only on the available surface area but more importantly on the extent of damaged cells. No significant differences in lipid digestibility were observed between raw and roasted almond samples.

The structural characteristics of ingested almonds and the mechanism(s) by which lipid is released from almond cells are of crucial importance, since these properties have a direct impact on physiological functions (e.g. gastric emptying, gut hormone secretion and microbial fermentation) and subsequently on the risks of developing certain conditions, such as cardiometabolic diseases. This has important implications for many other plant foods where structurally intact dietary fibre slows down and/or restricts energy release, digestion and absorption. The concept of an encapsulation mechanism could be employed in the design of novel food ingredients and foods targetted at consumers needing to reduce energy intake and improve body weight management.

## Figures and Tables

**Table 1:** Percentage of free fatty acid (FFA) released and initial reaction rate (μmol/min) for lipolysis of emulsions and unemulsified oils with pancreatin. Values are presented as means ± SEM (*n* = 3).

	Particle size (*d*_4,3_ in μm)^a^	FFA (%) at 60 min	Initial reaction rate (μmol/min)
Tributyrin emulsion	3.9 ± 0.2	99.1 ± 2.1	401 ± 21.5
Triolein emulsion	2.9 ± 0.1^b^^,^^c^	70.9 ± 2.7^b^^,^^c^	78.1 ± 3.1^b^^,^^c^
Raw almond oil emulsion	3.2 ± 0.0^b^^,^^c^	67.8 ± 2.7^b^^,^^c^	71.3 ± 6.3^b^^,^^c^
Roasted almond oil emulsion	3.4 ± 0.3^b^^,^^c^	70.4 ± 3.1^b^^,^^c^	78.1 ± 4.4^b^^,^^c^
Raw almond oil	ND	19.3 ± 0.9^b^	31.8 ± 2.1^b^
Roasted almond oil	ND	20.2 ± 0.6^b^	32.1 ± 4.2^b^

ND = Not Determined.

a Values for particle size are expressed as *d*_4,3_, which is the volume mean diameter ($d_{4\text{,}3}=\frac{\sum n_{i}d_{i}^{4}}{\sum n_{i}d_{i}^{3}}$).

b Statistically significant differences compared with tributyrin emulsion (*P* < 0.05).

c Statistically significant differences compared with almond oil, both raw and roasted (*P* < 0.05).

**Table 2:** Initial reaction rates (μmol/min) for lipolysis and total FFA production at 60 min of milled (particle size from <250 to 2000 μm) and chewed, raw and roasted almonds. FFA at 60 min and lipolysis rate values are presented as means ± SEM (*n* = 3).

	Particle size range (μm)	Predicted lipid released (%)^b^	FFA (%) at 60 min	Initial reaction rate (μmol/min)
Raw almonds	1000–2000	8.5	44.2 ± 5.3^c^	41.3 ± 3.7^c^
	500–1000	16.0	55.7 ± 1.6^c^	49.7 ± 3.6^c^
	250–500	30.0	60.9 ± 1.2	50.6 ± 2.7
	<250	39.0	63.9 ± 1.6	58.6 ± 1.6
	Cells	0.0^a^	31.2 ± 2.3^c^	18.0 ± 1.7^c^
	Chewed	8.5^b^	56.0 ± 5.2	64.0 ± 3.5

Roasted almonds	1000–2000	8.5	39.3 ± 1.1^c^	34.1 ± 1.5^c^
	500–1000	16.0	48.2 ± 2.2^c^	39.1 ± 3.1^c^
	250–500	30.0	55.4 ± 1.4	41.7 ± 3.0^c^
	<250	39.0	59.7 ± 0.8	51.3 ± 2.0^c^
	Cells	0.0^a^	31.4 ± 2.9^c^	24.8 ± 3.1^c^
	Chewed	11.3^b^	58.2 ± 1.2	64.6 ± 0.1

a Theoretically, the predicted lipid release values (see below) for cells should be zero; however, cell preparations contained some free lipid since a small number of cells were ruptured.

b Values were predicted from a theoretical model, using particle size data of milled almonds (raw and roasted); for details of the method see Grundy et al. (2015) and Grassby et al. (2014).

c Significant difference between milled particles and chewed samples (*P* < 0.05).
